# Child Abuse: The Consequence of an Undiagnosed Giant Olfactory Groove Meningioma?

**DOI:** 10.7759/cureus.13582

**Published:** 2021-02-27

**Authors:** Shoeb B Lallani, Dylan Adams, Hayley Doan, Emily Trieu, Ninh Doan

**Affiliations:** 1 Medicine, University of Alabama at Birmingham School of Medicine, Birmingham, USA; 2 Neurosurgery, University of Vermont Larner College of Medicine, Burlington, USA; 3 Medicine, University of South Alabama, Mobile, USA; 4 Medicine, Pleasant Grove High School, Elk Grove, USA; 5 Neurosurgery, Baptist Medical Center South, Montgomery, USA

**Keywords:** neurosurgery, meningioma, psychology

## Abstract

Olfactory groove meningiomas are slow-growing tumors that manifest with headaches, changes in vision, and personality changes. The anatomic location of these tumors makes psychiatric disturbances more common early in the stage of tumors than focal neurological deficits. The case study here describes a unique instance of an undiagnosed giant olfactory groove meningioma in a young mother who was charged with a felony of aggravated child abuse for the death of her toddler daughter. The patient underwent gross tumor resection and radiation therapy, which halted the visual decline, resolved the frontal headaches, and the patient showed improved mood. In this patient, the insidious onset of personality changes without obvious focal neurologic deficits until late as well as a history of depression likely contributed to the delayed diagnosis. Failure to notice these initial behavioral manifestations in these patients allows for further psychiatric and cognitive decline, which can result in devastating social consequences.

## Introduction

Meningiomas are benign, slow-growing tumors that originate from arachnoid cap cells and account for approximately 35% of all primary brain tumors. They have been reported to be more common in adult females than males [1]. Meningiomas can be found in many locations, including the olfactory groove. Olfactory groove meningiomas (OGMs) originate from the anterior cranial base, including the cribriform plate and planum sphenoidale [2]. They account for approximately 8-13% of intracranial meningiomas [3]. These tumors cause progressive compression of the frontal lobes with posterior projection towards the sella turcica. If large enough, the optic nerves and chiasm may be compressed. Headaches, anosmia, visual impairment, and personality changes are symptoms that have been commonly reported. Given the anatomic location of OGMs, there may be prolonged psychiatric symptoms before the presentation of focal neurologic deficits. The lack of discernable neurological symptoms makes these tumors among the largest intracranial tumors at presentation [4]. Here, we present a case of giant OGM presenting with bilateral visual impairments and prominent psychiatric manifestations that likely contributed to criminal behavior.

## Case presentation

A 28-year-old female with chronic depression, managed with Zoloft, presented to a local clinic with a seven-month history of bilateral visual disturbances and headaches. The patient was recently charged with felony aggravated child abuse, resulting in the death of an infant. On examination, she was found to have bilateral papilledema and was subsequently transferred to our emergency department for further evaluation. On history, she reported complete loss of vision on the left side with a severe decrease in acuity on the right. She stated that her visual deficits began approximately seven months prior and had been progressively declining. Additionally, she described a waxing and waning frontal headache for the past two months. On psychiatric questioning, she disclosed that she contemplated drowning herself in a bathtub. She was placed on one-to-one observation for suicide precautions.

On physical exam, the patient demonstrated a left afferent pupillary defect with complete vision loss. The right visual field was grossly intact but with severely decreased visual acuity, with the patient only able to see shapes and light. Extraocular movements were intact bilaterally. No other neurologic deficits were noted. Montreal Cognitive Assessment (MOCA) was 16/22 (unable to test vision) with particular deficits in short-term memory and serialization. Neuropsychological exam demonstrated impairment of alternating sequence testing Luria’s test, phonemic word fluency/FAS testing, digit span testing, and abstract thinking on subordinate categorization.

**Figure 1:**
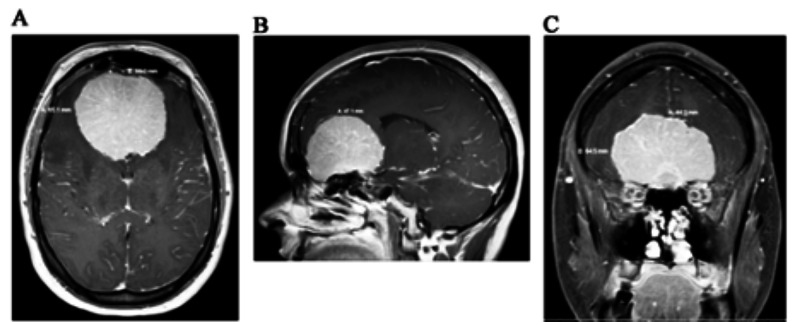
Pre-Op MRI MRI of the brain with contrast enhanced T1 (A) axial, (B) saggital, and (C) coronal reveals an enhancing 6.0 x 6.5 x 4.7 cm homogenous enhanced extra-axial mass consistent a giant olfactory groove meningioma.

An emergent computerized tomography (CT) without contrast suggested a giant planum sphenoidale meningioma measuring 6.2 x 5.9 x 4.0 cm. Surrounding vasogenic edema effaced the gray-white junction involving the bilateral frontal lobes and temporal horns. Mass effect was noted on the lateral ventricles with superior displacement of the genu of the corpus callosum. There was osseous remodeling of the cribriform plate and sphenoid bone with inferior depression of the cribriform plate (Figure 1).

**Figure 2:**
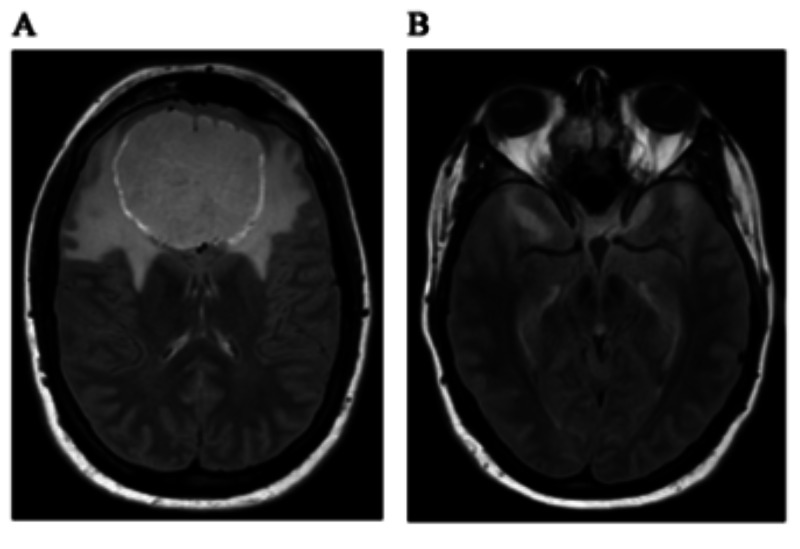
Pre-Op CT Contrast-enhanced T2 flair magnetic resonance imaging revealing significant cerebral edema involving the bifrontal lobes (A) and temporal horns (B).

Computed tomographic angiography (CTA) of the brain and neck revealed mass effect with displacement of the anterior cerebral arteries superiorly. Magnetic resonance imaging (MRI) of the brain with and without intravenous gadolinium further delineated an enhancing 6.0 x 6.5 x 4.7 cm T1 isointense and T2 hyperintense OGM with internal hemorrhage. There was surrounding T2/flair prolongation in the bifrontal lobes and temporal horns involving the white matter and cortex, representing edema. Posterior displacement of the anterior commissure and anterior communicating artery was evident. The pituitary gland was flattened (Figure 2).

**Figure 3:**
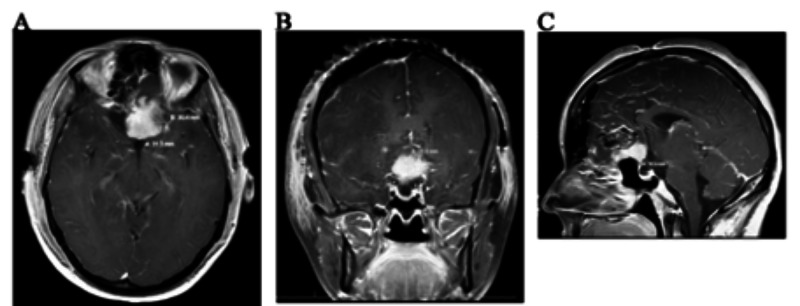
Post-Op CT Post-operative contrast-enhanced T1 axial (A), coronal (B), and sagittal (C) magnetic resonance imaging with residual mass in the anterior cranial fossa superior to the sella turcica.

The patient underwent angiography and embolization of the anterior and posterior ethmoidal branches of the right and left ophthalmic arteries. This was followed by a right frontal craniotomy with gross tumor resection (Figure 3). Surgical tissue pathology confirmed the diagnosis as a WHO grade I meningioma.

At one-month follow-up, the patient stated that her vision had progressively worsened since surgery. She could only see light out of her right eye at that time. At the three-month follow-up, the patient had completed radiation therapy, expressed that her vision had remained stable, and her mood and affect had shown significant improvement.

## Discussion

Olfactory groove meningiomas are slow-growing tumors that arise from the anterior cranial base. These tumors cause progressive compression of the frontal lobes with posterior projection towards the sella turcica. Headaches, anosmia, visual impairment, and personality changes are common presenting symptoms. Given the anatomic location of OGMs, there may be prolonged psychiatric and/or cognitive symptoms before the presentation of focal neurologic deficits [4]. These initial psychiatric and cognitive symptoms may be overlooked or attributed to substance abuse, mental illness, or cognitive impairment disorders such as dementia.

The statements from the family about the patient are consistent with the observation that she had an insidious and progressive change in personality, attention, and cognition over multiple years. Per interview with a criminal case investigator, she underwent a drastic change in behavior approximately two years prior to her arrest. She was reported to have been a well-kept and tidy individual before moving to the current state. In fact, her husband questioned the charges, explaining that she used to go on mission trips to support women and children suffering in broken homes. “Fighting against abuse and neglect was part of what we did. She was so dedicated to the cause,” he said. Unlike her past self, at the time of arrest, the patient’s home was filled with the odor of human excrements and littered with rotting food, garbage, dirty diapers, and used condoms.

TheFrontal Lobe Paradox is seen in patients with lesions in the frontal lobe. These patients exhibit significant impairment with tasks of daily living which include multitasking, judgement, and complex behavioral organization. Interestingly, these patients show unimpaired performance on conventional neuropsychological and intelligence exams. Patients also demonstrate a lack of insight regarding their condition because the self-monitoring center exists in the damaged frontal lobe. Often following the behavioral consequences, continued growth of the tumor can eventually compromise the optic nerves and chiasm with the potential for irreversible damage. The delay in recognition of our patient’s behavioral and cognitive manifestations of an OGM likely allowed for it to increase in size to > 6 cm in diameter at presentation and compromise the optic nerves and chiasm. However, identifying OGMs before they grow to large sizes has historically been challenging. In fact, a study by Jung and colleagues of 59 patients with OGMs revealed that greater than 70% presented with a tumor diameter ≥ 4 cm. This study classified OGMs by size on neuroimaging: small (0-2 cm in diameter), medium (2-4 cm diameter), large (4-6 cm diameter), and giant (> 6 cm in diameter) [5,6].

Recent pregnancy in our patient could also have played a role in the growth of her OGM. Studies have shown tremendous growth of meningiomas during pregnancy and in the postpartum period. Although the definitive cause is unknown, experts hypothesize a role of progesterone in this phenomenon due to the high rate of progesterone receptors in meningiomas and the drastic increase in progesterone levels during pregnancy [7,8]. Furthermore, progesterone is known to increase cerebrovascular inflammation and dilation, corresponding to increased intracellular and extracellular edema seen in meningiomas during pregnancy [9,10,11]. Given our patient’s gravidity and recent pregnancy, an acceleration of her OGM size and symptoms can be hypothesized. However, our patient’s history of depression and the possibility of pituitary dysfunction cannot be entirely ruled out as contributories to her criminal behavior.

The historical, observational, and objective findings of this case are highly suggestive of frontal lobe dysfunction, which contributed to the death of her daughter. If these observational and behavioral changes had been considered during the seven-month incarceration, a diagnosis could have been made sooner.

## Conclusions

OGMs are generally difficult to diagnose early. However, patients with drastic personality changes with or without neurological deficit should undergo prompted medical evaluation. Early recognition of intracranial masses can help to prevent irreversible anatomical damage and deter drastic behavioral changes in patients.

## References

[1] Marosi C, Hassler M, Roessler K, Reni M, Sant M, Mazza E, Vecht C (2008). Meningioma. Crit Rev Oncol Hematol.

[2] Pepper JP, Hecht SL, Gebarski SS, Lin EM, Sullivan SE, Marentette LJ (2011). Olfactory groove meningioma: discussion of clinical presentation and surgical outcomes following excision via the subcranial approach. Laryngoscope.

[3] Nakamura M, Struck M, Roser F, Vorkapic P, Samii M (2007). Olfactory groove meningiomas: clinical outcome and recurrence rates after tumor removal through the frontolateral and bifrontal approach. Neurosurgery.

[4] Ciurea AV, Iencean SM, Rizea RE, Brehar FM (2012). Olfactory groove meningiomas: a retrospective study on 59 surgical cases. Neurosurg Rev.

[5] Duncan J, Emslie H, Williams P, Johnson R, Freer C (1996). Intelligence and the frontal lobe: the organization of goal-directed behavior. Cogn Psychol.

[6] Jung JJ, Warren FA, Kahanowicz R (2012). Bilateral visual loss due to a giant olfactory meningioma. Clin Ophthalmol.

[7] Bickerstaff ER, Small JM, Guest IA (1958). The relapsing course of certain meningiomas in relation to pregnancy and menstruation. J Neurol Neurosurg Psychiat.

[8] Michelsen JJ, New PF (1969). Brain tumour and pregnancy. J Neurol Neurosurg Psychiat.

[9] Hatiboglu MA, Cosar M, Iplikcioglu AC, Ozcan D (2008). Sex steroid and epidermal growth factor profile of giant meningiomas associated with pregnancy. Surg Neurol.

[10] Krause DN, Duckles SP, Pelligrino DA (2006). Influence of sex steroid hormones on cerebrovascular function. J Appl Physiol.

[11] Lusis EA, Scheithauer BW, Yachnis AT, Fischer BR, Chicoine MR, Paulus W, Perry A (2012). Meningiomas in pregnancy: a clinicopathologic study of 17 cases. Neurosurgery.

